# Keeping it simple: public health diplomacy along the China–Laos railway

**DOI:** 10.3389/phrs.2026.1610119

**Published:** 2026-09-28

**Authors:** Emily Ying Yang Chan, Dong Dong, Chun-ying Leung

**Affiliations:** 1 Collaborating Centre for Oxford University and the Chinese University of Hong Kong for Disaster and Medical Humanitarian Response, Shatin, Hong Kong SAR, China; 2 The Chinese University of Hong Kong the Jockey Club School of Public Health and Primary Care, Shatin, Hong Kong SAR, China; 3 GX Foundation, Hong Kong, Hong Kong SAR, China

**Keywords:** dengue fever, disaster risk reduction, health emergencies, implementation, public health diplomacy

## Introduction: from high-level commitments to local implementation

Health diplomacy, the mobilization of health issues to influence foreign policy and interstate relations, is increasingly important: public health problems frequently intersect with geopolitical competition, economic integration and infrastructural development. Infectious diseases move through transport networks and across borders, while the authority and resources needed to address them are distributed among differing stakeholders. This pattern raises a practical question: how can political commitments become effective, locally legitimate interventions when no institution has sufficient capacity to act alone?

Health diplomacy has traditionally been examined through global health diplomacy (“GHD”), which concerns the negotiation of health interests through foreign policy, international relations and global governance [1, 2]. This literature explains how states establish agreements, mobilize resources and incorporate health into strategic agendas. However, health diplomacy increasingly depends on sub-state and non-state actors working in hospitals, communities, transport systems and emergencies. Public health diplomacy (“PHD”) offers a lens for examining the practical negotiations through which public health capacity and resilience are built [3].

China’s Belt and Road Initiative (“BRI”), a global infrastructure and connectivity programme, is a case in point. Within the BRI, an initiative known as the Health Silk Road (“HSR”) seeks to strengthen global health cooperation, improve healthcare infrastructure and expand China’s influence in international health governance. Transport corridors facilitate trade but also create pathways for disease transmission, requiring cooperation among health authorities, infrastructure operators and communities. The HSR thus illustrates how infrastructure, mobility and health security are intertwined [4, 5]. Its outcomes, however, depend on specific epidemiological risks and institutional contexts.

This commentary examines these socio-political dynamics through GX Foundation’s work along the China–Laos Railway and its later flood response in northern Laos against the backdrop of the HSR. Established in Hong Kong [6] in 2018, GX provides international medical and humanitarian assistance through partnerships that emphasize “people-to-people connectivity” [7]. Its boundary-spanning position demonstrates how non-state actors connect diplomatic commitments with operational expertise. We conclude that GHD secures high-level commitments, while PHD makes them workable and durable. Although conceptually distinct, they are practically complementary.

## Preventing dengue along a transnational railway corridor

The China–Laos Railway is a 1,035-km BRI corridor connecting Kunming and Vientiane through dengue-endemic areas. While advancing economic integration, it creates a shared epidemiological space in which mosquitoes, infected travellers and disease risks cross national boundaries. Managing this risk requires translating national commitments into coordinated practices across stations and communities.

Since 2020, GX has worked under Lao national and provincial health authorities with the Laos–China Railway Company, railway personnel and local stakeholders to embed dengue prevention in railway operations through a “Health Emergency and Disaster Risk Management” approach [8].

The initiative illustrates PHD as the cross-border mobilization of non-state and sub-state actors. Formal China–Laos engagement provided legitimacy and communication but left open how prevention would operate at individual stations. GX and the railway company filled this implementation gap by determining where equipment should be installed, who would maintain it, how staff would participate and how messages would be disseminated. The company provided infrastructural access, operational knowledge and logistics; Chinese and Lao personnel adapted interventions to station layouts and exposure patterns; and passengers and communities participated through risk communication and preventive activities.

Implementation required negotiation rather than simple equipment delivery. Sites were selected according to passenger volume, existing coverage and exposure to cross-border movements. Measures were adapted to station layouts, staff routines and electricity availability. Monitoring, for example, showed that posters were useful for staff who saw them repeatedly but less so for transient passengers.

By July 2024, 871 mosquito-killing lamps had been deployed across all 29 stations — 511 in China and 360 in Laos—in waiting halls, ticketing areas, washrooms, platforms, offices and staff accommodation. Monitoring at six locations, together with 60 interviews and 1,243 staff survey responses, informed maintenance and adaptation [8]. High satisfaction and preventive behaviours showed participation beyond equipment coverage. No dengue infections were reported among railway staff during the following 12 months, although this observational result cannot establish causation. Strong acceptance of the programme also contributed to the partnership’s two-year renewal in 2025 [9].

## Repurposing diplomatic infrastructure during an emergency

The programme’s relationships proved valuable during the September 2024 response to Typhoon Yagi. GX’s capacity to act in northern Laos arose not from a linear expansion of dengue-control activities but from existing, overlapping forms of health cooperation.

After severe flooding in Luang Namtha, assessments by GX and local health partners identified increased post-flood dengue risk. Flooding also damaged Namtha District Hospital’s laboratory facilities, weakening local diagnosis and referral. Under provincial and national health authorities, GX launched the four-month Project X, supplying vector-control materials and laboratory equipment to restore diagnostic and disease-control capacity [8].

Rather than creating an emergency partnership from scratch, Project X mobilized existing relationships. Authorities set priorities and retained oversight; hospital personnel identified the support required to resume testing and referrals; and GX mobilized supplies, diagnostic equipment and implementation support through Chinese and Lao networks. Local health workers and communities extended surveillance and prevention beyond the hospital, expanding diagnostic coverage from approximately 20–78 villages [8].

GX acted as a boundary-spanning intermediary connecting authorities, hospitals and communities with transport, logistics and other stakeholders under the legitimacy provided by authorities. Health facilities identified clinical needs, while local partners contributed contextual knowledge. Relationships formed through vector control and transport operations became what we call “diplomatic infrastructure”, producing immediate outputs and latent emergency-response capacity. Effective implementation depended both on the use of the railway corridor to deliver assistance and also on the resilience of the networks surrounding it.

## Conclusion: implications for GHD and PHD

Both cases show PHD as the practical, relationship-based complement to GHD. The railway programme was preventive and corridor-based, focusing on sustained risk management within commercial infrastructure. The flood response was reactive and geographically broader in addressing disrupted diagnostic capacity. It relied on trust and coordination developed through earlier programmes. Routine cooperation thus helped create “diplomatic infrastructure” that became rapidly repurposed during emergencies.

Three implications follow. First, implementation is a distinct diplomatic site: negotiation continues as agreements are interpreted, adapted and operationalized. Second, non-state actors are boundary-spanning intermediaries among health authorities, infrastructure operators, diplomatic institutions and communities. Their value lies not only in delivering resources but also in translating priorities and building local ownership of problems [10]. Third, evaluations should extend beyond immediate outputs to include relationships, trust and coordination capacities.

PHD should not be conflated with mere communication or programme coordination. It becomes necessary when actors must reconcile different interests, mandates, expertise and control over scarce resources. Negotiations may concern infrastructure access, responsibilities, intervention adaptation and local knowledge. Implementation is thus far from being a technical stage following agreement, but instead a setting where commitments are interpreted, adapted or resisted.

GHD and PHD should remain conceptually distinct but practically complementary. HSR conjoins health objectives with national strategy and international relations, while the GX–China–Laos Railway experience shows how these ambitions become practice through shared problem-solving.

This complementarity requires equity and accountability. NGO–enterprise partnerships work best when roles are clear, financing supports continuity and activities strengthen public institutions. Equitable involvement of local authorities and communities in decisions and implementation promotes ownership and responsiveness. PHD should thus be understood as ethical, accountable diplomacy that strengthens public health resilience.
